# Supplementary material for: Development of Androgen-Antagonistic Coumarinamides with a Unique Aromatic Folded Pharmacophore
Source: Int J Mol Sci. 2020 Aug 4;21(15):5584. doi: 10.3390/ijms21155584 (PMC7432827; doi:10.3390/ijms21155584)
Supplement: Supplementary file 1 [file ijms-21-05584-s001.pdf]

Supplementary information

Development of novel androgen antagonistic coumarinamides bearing unique aromatic folded structure

Hitomi Koga,[a] Mai Negishi,[a] Marie Kinoshita,[a] Shinya Fujii,[b] Shuichi Mori,[b] Mari Ishigami-Yuasa,[b] Emiko Kawachi,[b] Hiroyuki Kagechika,[b,]* and Aya Tanatani[a,]*

[a]*Department of Chemistry, Faculty of Science, Ochanomizu University, 2-1-1 Otsuka, Bunkyo-ku, Tokyo 112-8610, Japan*

[b]*Institute of Biomaterials and Bioengineering, Tokyo Medical and Dental University (TMDU), 2-3-10 Kanda-Surugadai, Chiyoda-ku, Tokyo 101-0062, Japan*

Compound data

Compound **7a**
^{1}H NMR (400 MHz, $CDCl_3$) δ 8.00 (1 H, br), 7.90 (2 H, d, *J* = 7.3 Hz), 7.71 (1 H, dd, *J* = 2.3, 8.0 Hz), 7.67 (1 H, d, *J* = 2.3 Hz), 7.59 (2 H, m), 7.54 (2 H, d, *J* = 7.8 Hz), 6.24 (1 H, d, *J* = 0.9 Hz), 2.44 (3 H, d, *J* = 0.9 Hz); Anal calcd for $C_{17}H_{13}NO_3$: C, 73.11, H, 4.69, N, 5.02, Found : C, 72.95, H, 4.64, N, 4.98.

Compound **7b**
^{1}H NMR (400 MHz, $CDCl_3$) δ 7.42 (1 H, d, *J* = 8.2 Hz), 7.33 (2 H, d, *J* = 6.9 Hz), 7.30 (1 H, t, *J* = 7.8 Hz), 7.21 (1 H, t, *J* = 7.8 Hz), 7.03 (1 H, d, *J* = 2.3 Hz), 6.94 (1 H, dd, *J* = 2.3, 8.0 Hz), 6.24 (1 H, d, *J* = 1.4 Hz), 3.54 (1 H, s), 2.37 (3 H, d, *J* = 0.9 Hz); ^{13}C NMR (150 MHz, $CDCl_3$) δ 170.6, 160.3, 154.0, 151.7, 148.0, 135.4, 130.3, 128.7, 128.1, 125.0, 122.6, 117.8, 114.8, 114.5, 38.3, 18.6; Anal calcd for $C_{18}H_{15}NO_3$ ·1/12 H_2O: C, 73.30, H, 5.19, N, 4.75, Found: C, 73.33, H, 5.10, N, 4.84.

Compound **8a**
^{1}H NMR (600 MHz, DMSO-*d*$_6$) δ 10.85 (1 H, s), 8.12 (2 H, d, *J* = 8.4 Hz), 8.05 (2 H, d, *J* = 8.4 Hz), 7.92 (1 H, s), 7.79 (1 H, d, *J* = 8.4 Hz), 7.74 (1 H, dd, *J* = 8.4 Hz, *J* = 1.8 Hz), 6.31 (1 H, s), 2.42 (3 H, s); ^{13}C NMR (150 MHz, DMSO-*d*$_6$): δ 160.4, 160.1, 153.6, 153.2, 142.2, 139.4, 132.9, 129.9, 128.3, 125.9, 116.1, 115.4, 112.6, 106.6, 18.1; Anal calcd for $C_{18}H_{12}N_2O_3$ ·1/4 H_2O: C, 69.67, H, 4.11, N, 9.03, Found: C, 69.85, H, 4.21, N, 9.24; HRMS calcd for MH^+: 305.0921, Found: 305.0930.

Compound **8b**
^{1}H NMR (600 MHz, $CDCl_3$) δ 7.52 (2 H, d, *J* = 8.4 Hz), 7.44 (1 H, d, *J* = 8.4 Hz), 7.42 (2 H, d, *J* = 8.4 Hz), 7.03 (1 H, d, *J* = 2.4 Hz), 7.90 (1 H, dd, *J* = 8.4 Hz, *J* = 1.8 Hz), 6.28 (1 H, s), 2.39(3 H, s); ^{13}C NMR (150 MHz, $CDCl_3$) δ 168.7, 160.1, 154.0, 151.6, 146.9, 139.7, 132.2, 129.3, 125.6, 122.7, 118.7, 118.0, 115.5, 114.8, 114.0, 38.4, 18.8; Anal calcd for $C_{19}H_{14}N_2O_3$ ·1/3H_2O: C, 69.72, H, 4.62, N, 8.56, Found: C, 69.84, H, 4.45, N, 8.40; HRMS calcd for MH^+: 319.1077, Found: 319.1069.

Compound **9a**
^{1}H NMR (600 MHz, DMSO-*d*$_6$) δ 10.94 (1 H, s), 8.39 (2 H, d, *J* = 6.9 Hz), 8.21 (2 H, d, *J* = 6.9 Hz), 7.93 (1 H, d, *J* = 2.4 Hz), 7.80 (1 H, d, *J* = 9.0 Hz), 7.75 (1 H, dd, *J* = 8.7 Hz, 2.1 Hz), 6.31 (1 H, s), 2.44 (3 H, s); ^{13}C NMR (150 MHz, DMSO-*d*$_6$): δ 164.5, 160.0, 153.5, 153.1, 149.4, 142.0, 140.1, 129.4, 125.9, 123.6, 116.3, 115.8, 112.8, 106.9, 18.0; Anal calcd for $C_{17}H_{12}N_2O_5$: C, 62.96, H, 3.73, N, 8.64, Found: C, 62.81, H, 3.94, N, 8.63; HRMS calcd for MH^+: 325.0819, Found: 325.0817.

Compound **9b**

^1H NMR (600 MHz, $CDCl_3$) δ 8.07 (2 H, d, *J* = 8.4 Hz), 7.49 (2 H, d, *J* = 8.4 Hz), 7.45 (1 H, d, *J* = 8.4 Hz), 7.04 (1 H, d, *J* = 2.4 Hz), 6.92 (1 H, d, *J* = 8.4 Hz), 3.55 (3 H, s), 2.38 (3 H, s); ^{13}C NMR (150 MHz, $CDCl_3$) δ 168.4, 160.1, 154.0, 151.6, 148.5, 146.8, 141.5, 129.7, 125.7, 123.6, 122.7, 118.8, 115.6, 114.9, 38.4, 18.8; Anal calcd for $C_{18}H_{14}N_2O_5 \cdot 1/6H_2O$: C, 63.23, H, 4.25, N, 8.19, Found: C, 63.46, H, 4.20, N, 8.16; HRMS calcd for MH^+: 339.0975, Found: 339.0973.

Compound **10a**
^1H NMR (600 MHz, DMSO-*d6*) δ 10.84 (1 H, s), 8.17 (2 H, d, *J* = 7.8 Hz), 7.94 (2 H, d, *J* = 9.0 Hz), 7.93 (1 H, s), 7.79 (1 H, d, *J* = 8.4 Hz), 7.75 (1 H, dd, *J* = 8.4 Hz, *J* = 1.8 Hz), 2.42 (3 H, s); ^{13}C NMR (150 MHz, DMSO-*d6*) δ 165.1, 160.1, 153.5, 153.1, 142.3, 138.3, 131.7 (q, *J* = 31.5 Hz), 128.9, 126.0, 125.6 (q, *J* = 3.0 Hz), 123.9 (q, *J* = 270.0 Hz), 116.3, 115.7, 112.7, 106.8, 18.1; Anal calcd for $C_{18}H_{12}F_3NO_3$: C, 62.25, H, 3.48, N, 4.03, Found: C, 62.07, H, 3.71, N, 4.16; HRMS calcd for MH^+: 348.0842, Found: 348.0839.

Compound **10b**
^1H NMR (600 MHz, $CDCl_3$) δ 7.49 (2 H, d, *J* = 8.4 Hz), 7.45 (2 H, d, *J* = 8.4 Hz), 7.45 (1 H, d, *J* = 8.4 Hz), 7.06 (1 H, d, *J* = 1.8 Hz), 6.92 (1 H, dd, *J* = 8.4 Hz, *J* = 2.4 Hz), 3.55 (3 H, s), 2.39 (3 H, s); ^{13}C NMR (150 MHz, $CDCl_3$) δ 169.2, 160.3, 154.0, 151.8, 147.3, 138.9, 132.1 (q, *J* = 31.5 Hz), 129.1, 125.5, 125.4 (q, *J* = 3.0 Hz), 123.6 (q, *J* = 271.5 Hz), 122.9, 118.5, 115.4, 114.8, 38.5, 18.1; Anal calcd for $C_{19}H_{14}F_3NO_3$: C, 63.16, H, 3.91, N, 3.88, Found: C, 63.17, H, 4.14, N, 3.83; HRMS calcd for MH^+: 362.0999, Found: 362.0989.

Compound **11a**
^1H NMR (600 MHz, DMSO-*d6*) δ 10.65 (1 H, s), 8.05 (2 H, d, *J* = 8.4 Hz), 7.92 (1 H, *J* = 3.6 Hz), 7.77 (1 H, d, *J* = 8.4 Hz), 7.74 (1 H, dd, *J* = 9.0 Hz, *J* = 1.8 Hz), 7.39 (2 H, t, *J* = 8.4 Hz), 2.41 (3 H, s); ^{13}C NMR (150 MHz, DMSO-*d6*) δ 165.6, 164.8 (d, *J* = 247.5 Hz), 160.6, 154.0, 153.6, 143.0, 131.3 (d, *J* = 1.5 Hz), 131.1 (d, *J* = 9.0 Hz), 126.3, 116.6, 116.0 (d, *J* = 21.0 Hz), 115.9, 113.0, 107.1, 18.5; Anal calcd for $C_{17}H_{12}FNO_3$: C, 68.68, H, 4.07, N, 4.71, Found: C, 68.40, H, 4.15, N, 4.77; HRMS calcd for MH^+: 298.0874, Found: 298.0867.

Compound **11b**
^1H NMR (600 MHz, $CDCl_3$) δ 7.43 (1 H, d, *J* = 8.4 Hz), 7.36-7.33 (2 H, m), 7.03 (1 H, d, *J* = 2.4 Hz), 6.93-6.88 (3 H, m), 6.26 (1 H, s), 3.53 (3 H, s), 2.38 (3 H, s); ^{13}C NMR (150 MHz, $CDCl_3$) δ 169.7, 163.7 (d, *J* = 250.5 Hz), 160.4, 154.0, 151.8, 148.0, 131.4 (d, *J* = 3.0 Hz), 131.2 (d, *J* = 9.0 Hz), 125.3, 122.7, 118.1, 115.5 (d, *J* = 22.5 Hz), 115.1, 114.6, 38.5, 18.8; Anal calcd for $C_{18}H_{14}FNO_3$: C, 69.45, H, 4.53, N, 4.50, Found: C, 69.21, H, 4.58, N, 4.42; HRMS calcd for MH^+: 312.1030, Found: 312.1025.

Compound **12a**
^1H NMR (600 MHz, DMSO-*d6*) δ10.69 (1 H, s), 8.00 (2 H, d, *J* = 8.4 Hz), 7.92 (1 H, d, *J* = 1.8 Hz), 7.77 (1 H, d, *J* = 8.4 Hz), 7.73 (1 H, dd, *J* = 8.4 Hz, *J* = 1.8 Hz), 7.63 (2 H, d, *J* = 8.4 Hz), 6.29 (1 H, s), 2.41 (3 H, s); ^{13}C NMR (150 MHz, DMSO-*d6*): δ 165.0, 160.1, 153.5, 153.2, 142.4, 136.9, 133.1, 131.2, 129.9, 128.8, 128.7, 125.9, 116.2, 115.5, 112.6, 106.7, 18.1; Anal calcd for $C_{17}H_{12}ClNO_3$: C, 65.08, H, 3.86, N, 4.46, Found: C, 64.89, H, 3.93, N, 4.38; HRMS calcd for MH^+: 314.0578, Found: 314.0569.

Compound **12b**
^1H NMR (600 MHz, $CDCl_3$) δ 7.43 (1 H, d, *J* = 8.4 Hz), 7.28 (2 H, d, *J* = 9.0 Hz), 7.19 (2 H, d, *J* = 9.0 Hz), 7.03 (1 H, d, *J* = 2.4 Hz), 7.91 (1 H, dd, *J* = 8.4 Hz, *J* = 1.8 Hz), 6.26 (1 H, s), 3.53 (3 H, s), 2.39 (3 H, s); ^{13}C NMR (150 MHz, $CDCl_3$): δ 169.6, 160.4, 154.0, 151.8, 147.8, 136.6, 133.7, 130.3, 128.6, 125.4, 122.8, 118.2, 115.2, 114.7, 38.5, 18.8; Anal calcd for $C_{18}H_{14}ClNO_3$: C, 65.96, H, 4.31, N, 4.27, Found: C, 65.69, H, 4.42, N, 4.21; HRMS calcd for MH^+: 328.0735, Found: 328.0734.

Compound **13a**
^1H NMR (600 MHz, DMSO-*d6*) δ 10.54 (1 H, s), 7.93 (1 H, s), 7.89 (2 H, t, *J* = 8.4 Hz), 7.76 (2 H, s), 7.36 (2 H, d, *J* = 7.8 Hz), 6.28 (1 H, s), 2.49 (3 H, s), 2.40 (3 H, s); ^{13}C NMR (150 MHz, DMSO-*d6*): δ

166.0, 160.2, 154.0, 153.3, 142.8, 142.2, 131.6, 129.1, 127.9, 125.9, 116.2, 115.3, 112.5, 106.6, 21.1, 18.1; Anal calcd for $C_{18}H_{15}NO_3$: C, 73.71, H, 5.15, N, 4.78, Found: C, 73.58, H, 5.15, N, 4.91; HRMS calcd for MH^+: 294.1125, Found: 294.1124.

Compound **13b**
^{1}H NMR (600 MHz, $CDCl_3$) δ 7.43 (1 H, d, *J* = 8.4 Hz), 7.23 (2 H, d, *J* = 7.8 Hz), 7.02 (1 H, s), 7.01 (1 H, d, *J* = 7.8 Hz), 6.95 (1 H, d, *J* = 8.4 Hz), 6.24 (1 H, s), 3.52 (3 H, s), 2.38 (3 H, s), 2.27 (3 H, s); ^{13}C NMR (150 MHz, $CDCl_3$) δ 170.8, 160.6, 153.9, 152.0, 148.4, 140.8, 132.4, 129.0, 128.9, 125.2, 122.6, 117.8, 114.9, 114.6, 38.4, 21.6, 18.8; Anal calcd for $C_{19}H_{17}NO_3 \cdot 1/9\ H_2O$: C, 73.71, H, 5.62, N, 4.52, Found: C, 73.79, H, 5.82, N, 4.52; HRMS calcd for MH^+: 308.1281, Found: 308.1275.

Compound **14a**
^{1}H NMR (600 MHz, DMSO-*d₆*) δ 10.47 (1 H, s), 7.97 (2 H, d, *J* = 6.9 Hz), 7.94 (1 H, s), 7.75 (1 H, s), 7.08 (2 H, d, *J* = 6.9 Hz), 6.27 (1 H, s), 3.84 (3 H, s), 2.41 (3 H, s); ^{13}C NMR (150 MHz, DMSO-d_6) δ 165.5, 162.3, 160.2, 153.6, 153.3, 142.9, 129.9, 126.4, 125.8, 116.1, 115.2, 113.8, 112.4, 106.5, 55.6, 18.1; Anal calcd for $C_{18}H_{15}NO_4$: C, 69.89, H, 4.89, N, 4.53, Found: C, 69.60, H, 4.92, N, 4.56; HRMS calcd for MH^+: 310.1074, Found: 310.1068.

Compound **14b**
^{1}H NMR (600 MHz, $CDCl_3$) δ 7.42 (1 H, d, *J* = 8.4 Hz), 7.30 (2 H, d, *J* = 8.4 Hz), 7.02 (1 H, d, *J* = 1.8 Hz), 6.93 (1 H, dd, *J* = 8.1 Hz, *J* = 1.8 Hz), 6.70 (2 H, d, *J* = 8.4 Hz), 6.24 (1 H, s), 3.75 (3 H, s), 3.52 (3 H, s), 2.38 (3 H, s); ^{13}C NMR (150 MHz, $CDCl_3$) δ 170.5, 161.2, 160.6, 153.9, 152.0, 148.7, 131.0, 127.3, 125.1, 122.6, 117.7, 114.8, 114.5, 113.5, 55.4, 38.5, 18.8; Anal calcd for $C_{19}H_{17}NO_4$: C, 70.58, H, 5.30, N, 4.33, Found: C, 70.28, H, 5.40, N, 4.28; HRMS calcd for MH^+: 324.1230, Found: 324.1222.

Compound **15a**
^{1}H NMR (600 MHz, DMSO-d_6) δ 10.80 (1 H, s), 8.43 (1 H, s), 8.26 (1 H, d, *J* = 7.8 Hz), 8.10 (1 H, d, *J* = 7.8 Hz), 7.92 (1 H, d, *J* = 1.8 Hz), 7.79 (1 H, d, *J* = 9.0 Hz), 7.78 (1 H, t, *J* = 7.8 Hz), 7.73 (1 H, dd, *J* = 9.0 Hz, *J* = 2.4 Hz), 2.42 (3 H, s); ^{13}C NMR (150 MHz, DMSO-d_6) δ 164.3, 160.1, 153.5, 153.2, 142.2, 135.5, 132.8, 131.5, 130.0, 126.0, 118.3, 116.2, 115.7, 112.8, 111.6, 106.8, 18.1; Anal calcd for $C_{18}H_{12}N_2O_3 \cdot 1/4H_2O$: C, 69.67, H, 4.11, N, 9.03, Found: C, 69.63, H, 4.05, N, 8.99; HRMS calcd for MH^+: 305.0921, Found: 305.0912.

Compound **15b**
^{1}H NMR (600 MHz, $CDCl_3$) δ 7.62-7.58 (3 H, m), 7.48 (1 H, d, *J* = 8.4 Hz), 7.37 (1 H, t, *J* = 7.8 Hz), 6.99 (1 H, d, *J* = 2.4 Hz), 6.96 (1 H, dd, *J* = 8.4 Hz, *J* = 1.8 Hz), 3.54 (3 H, s), 2.37 (3 H, s); ^{13}C NMR (600 MHz, DMSO-d_6) δ 168.3, 160.2, 154.0, 151.7, 147.0, 136.8, 133.8, 133.0, 132.2, 129.4, 125.8, 122.5, 118.7, 118.0, 115.5, 114.9, 112.7, 38.6, 18.8; Anal calcd for $C_{19}H_{14}N_2O_3$: C, 71.69, H, 4.43, N, 8.80, Found: C, 71.45, H, 4.64, N, 8.71; HRMS calcd for MH^+: 319.1077, Found: 319.1068.

Compound **16a**
^{1}H NMR (600 MHz, DMSO-d_6) δ 10.83 (1 H, s), 8.31 (1 H, s), 8.28 (1 H, d, *J* = 8.4 Hz), 8.00 (1 H, d, *J* = 8.4 Hz), 7.93 (1 H, d, *J* = 2.4 Hz), 7.81 (1 H, t, *J* = 7.8 Hz), 7.79 (1 H, d, *J* = 9.0 Hz), 7.74 (1 H, dd, *J* = 9.0 Hz, *J* = 2.4 Hz), 6.31 (1 H, s), 2.42 (3 H, s); ^{13}C NMR (150 MHz, DMSO-d_6) δ 164.6, 160.0, 153.5, 153.0, 142.2, 135.3, 132.0, 129.8, 129.2 (q, *J* = 31.5 Hz), 128.5 (q, *J* = 3.5 Hz), 125.8, 124.4 (q, *J* = 4.5 Hz), 123.9 (q, *J* = 270.0 Hz), 116.3, 115.6, 112.7, 106.9, 18.0; Anal calcd for $C_{18}H_{12}F_3NO_3$: C, 62.23, H, 3.48, N, 4.03, Found: C, 62.09, H, 3.63, N, 4.05; HRMS calcd for MH^+: 348.0842, Found: 348.0845.

Compound **16b**
^{1}H NMR (600 MHz, $CDCl_3$) δ 7.63 (1 H, s), 7.56 (1 H, d, *J* = 7.8 Hz), 7.48 (1 H, d, *J* = 7.8 Hz), 7.46 (1 H, d, *J* = 9.0 Hz), 7.34 (1 H, t, *J* = 7.8 Hz), 7.03 (1 H, d, *J* = 2.4 Hz), 6.95 (1 H, dd, *J* = 8.4 Hz, *J* = 1.2 Hz), 3.55 (3 H, s), 2.38 (3 H, s); ^{13}C NMR (150 MHz, $CDCl_3$) δ 169.1, 160.3, 154.0, 151.8, 147.4, 136.1, 132.0, 130.8 (q, *J* = 33.0 Hz), 128.8, 127.1 (q, *J* = 3.0 Hz), 125.9 (q, *J* = 4.5 Hz), 125.5, 123.6

(q, J = 271.5 Hz), 122.7, 118.4, 115.3, 114.8, 38.5, 18.8; Anal calcd for $C_{19}H_{14}F_3NO_3$: C, 63.16, H, 3.91, N, 3.88, Found: C, 63.05, H, 4.00, N, 3.85; HRMS calcd for MH^+: 362.0999, Found: 362.0990.

Compound **17a**
^{1}H NMR (600 MHz, DMSO-d_6) δ 10.68 (1 H, s), 7.92 (1 H, d, J = 2.4 Hz), 7.83 (1 H, d, J = 7.2 Hz), 7.79 (1 H, d, J = 9.2 Hz), 7.78 (1 H, d, J = 8.4 Hz), 7.74 (1 H, dd, J = 9.0 Hz, J = 1.8 Hz), 7.61 (1 H, q, J = 7.8 Hz), 7.48 (1 H, td, J = 8.4 Hz, J = 2.4 Hz), 2.42 (3 H, s); ^{13}C NMR (150 MHz, DMSO-d_6) δ 164.8 (d, J = 1.5 Hz), 162.0 (d, J = 243.0 Hz), 160.1, 153.5, 153.2, 142.3, 136.7 (d, J = 7.5 Hz), 130.8 (d, J = 9.0 Hz), 125.9, 124.2 (d, J = 3.0 Hz), 119.0 (d, J = 21.0 Hz), 116.2, 115.6, 114.8 (d, J = 22.5 Hz), 112.7, 106.8, 18.1; Anal calcd for $C_{17}H_{12}FNO_3$: C, 68.68, H, 4.07, N, 4.71, Found: C, 68.45, H, 4.25, N, 4.65; HRMS calcd for MH^+: 298.0874, Found: 298.0865.

Compound **17b**
^{1}H NMR (600 MHz, $CDCl_3$) δ 7.45 (1 H, d, J = 8.4 Hz), 7.21-7.17 (1 H, m), 7.09 (1 H, dt, J = 7.8 Hz, J = 1.2 Hz), 7.60 (1 H, dt, J = 8.4 Hz, J = 1.2 Hz), 7.03 (1 H, d, J = 1.8 Hz), 7.00 (1 H, tdd, J = 8.4 Hz, J = 2.4 Hz, J = 1.2 Hz), 6.95 (1 H, dd, J = 8.4 Hz, J = 2.4 Hz), 6.26 (1 H, s), 3.53 (3 H, s), 2.39 (3 H, s); ^{13}C NMR (150 MHz, $CDCl_3$) δ 169.3 (d, J = 3 Hz), 162.2 (d, J = 246.0 Hz), 160.4, 153.9, 151.8, 147.6, 137.5 (d, J = 7.5 Hz), 130.1 (d, J = 7.5 Hz), 125.4, 124.5 (d, J = 3.0 Hz), 122.6, 118.3, 117.6 (d, J = 21.0 Hz), 115.9 (d, J = 22.5 Hz), 115.2, 114.7, 38.5, 18.8; Anal calcd for $C_{18}H_{14}FNO_3$: C, 69.45, H, 4.53, N, 4.50, Found: C, 69.16, H, 4.80, N, 4.57; HRMS calcd for MH^+: 312.1030, Found: 312.1025.

Compound **18a**
^{1}H NMR (600 MHz, DMSO-d_6) δ 10.71 (1 H, s), 8.03 (1 H, t, J = 1.8 Hz), 7.93 (1 H, dt, J = 7.8 Hz, J = 1.2 Hz), 7.92 (1 H, d, J = 2.4 Hz), 7.77 (1 H, d, J = 8.4 Hz), 7.74 (1 H, dd, J = 8.4 Hz, J = 1.8 Hz), 7.69 (1 H, d, J = 8.4 Hz), 7.59 (1 H, t, J = 7.8 Hz), 2.42 (3 H, s); ^{13}C NMR (150 MHz, DMSO-d_6) δ 164.7, 160.1, 153.5, 153.2, 142.3, 136.4, 133.3, 131.9, 130.6, 127.6, 126.8, 125.9, 116.2, 115.6, 112.7, 106.8, 18.1; Anal calcd for $C_{17}H_{12}ClNO_3$: C, 65.08, H, 3.86, N, 4.46, Found: C, 65.05, H, 3.98, N, 4.48; HRMS calcd for MH^+: 314.0578, Found: 314.0571.

Compound **18b**
^{1}H NMR (600 MHz, $CDCl_3$) δ 7.45 (1 H, d, J = 8.4 Hz), 7.40 (1 H, t, J = 1.8 Hz), 7.27 (1 H, dt, J = 7.2 Hz, J = 2.4 Hz), 7.13 (3 H, s), 7.04 (1 H, d, J = 2.4 Hz), 6.95 (1 H, dd, J = 8.4 Hz, J = 1.8 Hz), 3.53 (1 H, s), 2.39 (3 H, s); ^{13}C NMR (150 MHz, $CDCl_3$): δ 169.3, 160.4, 154.0, 151.8, 147.5, 137.2, 134.5, 130.6, 129.6, 129.0, 126.8, 125.4, 122.7, 118.3, 115.2, 114.7, 38.5, 18.8; Anal calcd for $C_{18}H_{14}ClNO_3$: C, 65.96, H, 4.31, N, 4.27, Found: C, 65.74, H, 4.44, N, 4.28; HRMS calcd for MH^+: 328.9735, Found: 328.0730.

Compound **18c**
^{1}H NMR (600 MHz, $CDCl_3$) δ 7.45 (1 H, d, J = 8.4 Hz), 7.39 (1 H, t, J = 1.8 Hz), 7.24 (1 H, t, J = 1.8 Hz), 7.14 (1 H, dt, J = 7.2 Hz, J = 1.8 Hz), 7.04 (1 H, d, J = 1.8 Hz), 6.93 (1 H, dd, J = 8.4 Hz, J = 2.4 Hz), 6.27 (3 H, s), 4.01 (3 H, q, J = 1.2 Hz), 2.39 (3 H, s), 1.24 (3 H, t, J = 7.2 Hz); ^{13}C NMR (150 MHz, DMSO-d_6) δ 168.8, 160.4, 154.0, 151.8, 146.1, 137.5, 134.4, 130.4, 129.5, 128.9, 126.7, 125.4, 123.7, 118.5, 115.5, 115.3, 45.8, 18.8, 13.2; Anal calcd for $C_{19}H_{16}ClNO_3$: C, 66.77, H, 4.72, N, 4.10, Found: C, 66.54, H, 4.94, N, 4.10; HRMS calcd for MH^+: 342.0891, Found: 342.0888.

Compound **18d**
^{1}H NMR (600 MHz, $CDCl_3$) δ 7.44 (1 H, d, J = 8.4 Hz), 7.38 (1 H, s), 7.25 (1 H, dt, J = 6.6 Hz, J = 1.8 Hz), 7.12 (1 H, s), 7.11 (1 H, d, J = 9.6 Hz), 7.04 (1 H, d, J = 2.4 Hz), 6.92 (1 H, dd, J = 8.4 Hz, J = 2.4 Hz), 3.91 (2 H, t, J = 1.8 Hz), 2.39 (3 H, s), 1.65 (2 H, m), 0.95 (3 H, t, J = 7.2 Hz); ^{13}C NMR (150 MHz, $CDCl_3$) δ 168.9, 160.4, 154.0, 151.8, 146.4, 137.6, 134.4, 130.4, 129.5, 128.9, 126.7, 125.4, 123.62, 118.4, 115.4, 115.2, 52.3, 21.2, 18.8, 11.5; Anal calcd for $C_{20}H_{18}ClNO_3$ (+1/8 H_2O): C, 67.30, H, 5.14, N, 3.91, Found: C, 67.13, H, 5.13, N, 3.98; HRMS calcd for MH^+: 356.1048, Found: 356.1041.

Compound **18e**

^{1}H NMR (600 MHz, MeOD) δ 7.56 (1 H, d, *J* = 8.4 Hz), 7.47 (1 H, d, *J* = 1.2 Hz), 7.33-7.26 (6 H, m), 7.24 (1 H, t, *J* = 6.6 Hz), 7.20 (1 H, t, *J* = 7.8 Hz), 7.12 (1 H, d, *J* = 2.4 Hz), 7.00 (1 H, dd, *J* = 8.4 Hz, *J* = 1.8 Hz), 6.26 (1 H, s), 5.22 (2 H, s), 2.37 (3 H, s); ^{13}C NMR (150 MHz, $CDCl_3$) δ 169.1, 160.4, 153.8, 151.8, 146.2, 137.2, 136.7, 134.5, 130.6, 129.5, 129.0, 128.9, 128.3, 128.0, 126.8, 125.3, 123.6, 118.5, 115.4, 115.3, 53.9, 18.8; Anal calcd for $C_{24}H_{18}ClNO_3$: C, 71.38, H, 4.49, N, 3.47, Found: C, 71.10, H, 4.63, N, 3.35; HRMS calcd for MH^+: 404.1048, Found: 404,1046.

Compound **18f**
^{1}H NMR (600 MHz, $CDCl_3$) δ 7.80 (3 H, m), 7.69 (1 H, s), 7.46 (3 H, m), 7.44 (1 H, d, *J* = 1.8 Hz), 7.31 (1 H, d, *J* = 8.4 Hz), 7.27 (1 H, d, *J* = 8.4 Hz), 7.20 (1 H, dt, *J* = 8.4 Hz, *J* = 1.8 Hz), 7.12 (1 H, t, *J* = 7.8 Hz), 6.99 (1 H, d, *J* = 2.4 Hz), 6.80 (1 H, dd, *J* = 8.4 Hz, *J* = 1.8 Hz), 6.21 (1 H, s), 5.33 (2 H, s), 2.32 (3 H, s); Anal calcd for $C_{28}H_{20}ClNO_3\cdot 1/6\ H_2O$: C, 73.60, H, 4.49, N, 3.07, Found: C, 73.74, H, 4.54, N, 3.13; HRMS calcd for MH^+: 454.1204, Found: 454.1199.

Compound **19a**
^{1}H NMR (600 MHz, DMSO-*d*$_6$) δ10.59 (1 H, s), 7.94 (1 H, s), 7.78 (1 H, s), 7.77-7.74 (3 H, br), 7.44 (1 H, s), 7.43 (1 H, s), 6.25 (1 H, s), 2.41 (3 H, d, *J* = 1.2 Hz)), 2.40 (3 H, s); ^{13}C NMR (150 MHz, DMSO-*d*$_6$): δ 166.3, 160.2, 153.6, 153.2, 142.7, 137.9, 134.5, 132.7, 128.5, 128.3, 125.9, 125.1, 116.2, 115.3, 112.5, 106.6, 21.0, 18.1; Anal calcd for $C_{18}H_{15}NO_3$: C, 54.41, H, 3.55, N, 3.52, Found: C, 54.15, H, 3.54, N, 3.43; HRMS calcd for MH^+: 294.1125, Found: 294.1116.

Compound **19b**
^{1}H NMR (600 MHz, $CDCl_3$) δ 7.43 (1 H, d, *J* = 8.4 Hz), 7.28 (1 H, s), 7.10 (1 H, d, *J* = 7.8 Hz), 7.05 (1 H, d, *J* = 7.8 Hz), 7.03 (1 H, d, *J* = 1.8 Hz), 7.00 (1 H, d, *J* = 1.3 Hz), 6.96 (1 H, dd, *J* = 8.4 Hz, *J* = 2.4 Hz), 6.24 (1 H, s), 3.53 (3 H, s), 2.38 (3 H, s), 2.27 (3 H, s); ^{13}C NMR (150 MHz, DMSO-*d*$_6$): δ 171.0, 160.6, 153.9, 151.9, 148.2, 138.3, 135.4, 131.2, 129.5, 127.9, 125.7, 125.1, 122.6, 117.9, 114.9, 114.6, 38.4, 21.6, 18.8; Anal calcd for $C_{19}H_{17}NO_3$: C, 74.25, H, 4.50, N, 4.53, Found: C, 74.27, H, 5.82, N, 4.61.

Compound **19c**
^{1}H NMR (600 MHz, $CDCl_3$) δ 7.42 (1 H, d, *J* = 8.4 Hz), 7.27 (1 H, t, *J* = 1.2 Hz), 7.08 (1 H, dt, *J* = 7.2 Hz, *J* = 1.8 Hz), 7.03 (1 H, d, *J* = 1.8 Hz), 7.03 (1 H, t, *J* = 7.2 Hz), 6.99 (1 H, d, *J* = 7.2 Hz), 6.94 (1 H, d, *J* = 1.8 Hz), 6.23 (1 H, s), 4.02 (3 H, q, *J* = 7.2 Hz), 3.38 (3 H, s), 2.26 (3 H, s), 1.24 (1 H, t, *J* = 7.2 Hz); ^{13}C NMR (150 MHz, $CDCl_3$) δ 170.5, 160.6, 153.9, 151.9, 146.8, 138.3, 135.7, 131.1, 129.5, 127.9, 125.7, 125.1, 123.6, 118.1, 115.5, 115.0, 45.7, 21.5, 18.8, 13.3; Anal calcd for $C_{20}H_{19}NO_3\cdot 1/9H_2O$: C, 74.23, H, 6.00, N, 4.33, Found: C, 74.25, H, 6.03, N, 4.40; HRMS calcd for MH^+: 322.1438, Found: 322.1431.

Compound **19d**
^{1}H NMR (600 MHz, $CDCl_3$) δ 7.41 (1 H, d, *J* = 8.4 Hz), 7.07 (1 H, d, *J* = 7.2 Hz), 7.30-7.10 (2 H, m), 7.02 (1 H, t, *J* = 7.8 Hz), 6.98 (1 H, d, *J* = 8.4 Hz), 6.93 (1 H, dd, *J* = 8.4 Hz, *J* = 1.2 Hz), 6.23 (1 H, d, *J* = 1.2 Hz), 3.92 (3 H, q, *J* = 7.2 Hz), 2.37 (3 H, s), 2.26 (3 H, s), 1.66 (2 H, sextet, *J* = 1.8 Hz), 0.94 (3 H, t, *J* = 7.2 Hz); ^{13}C NMR (150 MHz, $CDCl_3$) δ 170.7, 160.6, 153.9, 151.9, 147.1, 138.2, 135.8, 131.0, 129.5, 127.9, 125.7, 125.1, 123.6, 118.0, 115.3, 114.9, 52.2, 21.5, 21.3, 18.8, 11.5; Anal calcd for $C_{21}H_{21}NO_3\cdot 1/4H_2O$: C, 73.88, H, 6.40, N, 4.10, Found: C, 74.15, H, 6.30, N, 4.04; HRMS calcd for MH^+: 336.1594, Found: 336.1590.

Compound **19e**
^{1}H NMR (600 MHz, $CDCl_3$) δ 7.33 (1 H, d, *J* = 8.4 Hz), 7.33 (1 H, s), 7.31-7.29 (4 H, m), 7.27 (1 H, s), 7.10 (1 H, d, *J* = 7.8 Hz), 7.05-7.03 (2 H, m), 6.94 (1 H, d, *J* = 2.4 Hz), 6.83 (1 H, dd, *J* = 8.4 Hz, *J* = 2.4 Hz), 6.21 (1 H, s), 5,18 (2 H, s), 2.34 (3 H, s), 2.27 (3 H, s); ^{13}C NMR (150 MHz, DMSO-*d*$_6$) δ 170.9, 160.6, 153.7, 151.9, 146.9, 138.3, 137.1, 135.4, 131.3, 129.6, 128.9, 128.2, 127.9, 127.8, 125.8, 125.0, 123.5, 118.0, 115.2, 115.0, 53.8, 21.5, 18.8; Anal calcd for $C_{25}H_{21}NO_3\cdot 1/7H_2O$: C, 74.94, H, 5.29, N, 3.47, Found: C, 74.66, H, 5.46, N, 3.56; HRMS calcd for MH^+: 384.1594, Found: 384.1587.

Compound **19f**

^{1}H NMR (600 MHz, $CDCl_3$) δ 7.82-7.76 (2 H, m), 7.80 (1 H, d, *J* = 8.4 Hz), 7.71 (1 H, s), 7.48-7.45 (3 H, m), 7.36 (1 H, s), 7.29 (1 H, d, *J* = 8.4 Hz), 7.10 (1 H, d, *J* = 7.2 Hz, *J* = 0.6 Hz), 7.07 (1 H, d, *J* = 8.4 Hz), 7.05 (1 H, t, *J* = 7.2 Hz), 6.99 (1 H, s), 6.83 (1 H, dd, *J* = 8.4 Hz, *J* = 2.4 Hz), 6.19 (1 H, s), 5.35 (2 H, s), 2.31 (3 H, s), 2.27 (3 H, s); ^{13}C NMR (150 MHz, $CDCl_3$) δ 171.0, 160.5, 153.7, 151.9, 146.8, 138.4, 135.4, 134.6, 133.4, 132.9, 131.3, 129.7, 128.8, 128.0, 127.9, 127.8, 127.2, 126.4, 126.2, 126.1, 125.8, 125.0, 123.5, 118.1, 115.2, 115.0, 53.7, 21.5, 18.7; Anal calcd for $C_{29}H_{23}NO_3 \cdot 1/7H_2O$: C, 79.80, H, 5.39, N, 3.21, Found: C, 79.85, H, 5.71, N, 3.34; HRMS calcd for MH^+: 434.1751, Found: 434.1742.

Compound **20a**

^{1}H NMR (600 MHz, DMSO-*d*$_6$) δ 10.60 (1 H, s), 7.93 (1 H, d, *J* = 1.2 Hz), 7.76-7.75 (2 H, m), 7.55 (1 H, d, *J* = 7.8 Hz), 7.50 (1 H, s), 7.47 (1 H, t, *J* = 7.8 Hz), 7.19 (1 H, d, *J* = 7.8 Hz), 7.29 (1 H, s), 3.84 (3 H, s), 2.42 (3 H, s); ^{13}C NMR (150 MHz, DMSO-*d*$_6$) δ 165.9, 160.1, 159.3, 153.6, 153.2, 142.6, 135.8, 129.8, 125.9, 120.1, 117.8, 116.2, 115.4, 113.1, 112.5, 106.7, 55.4, 18.1; Anal calcd for $C_{18}H_{15}NO_4$: C, 69.89, H, 4.89, N,4.53, Found: C, 69.62, H, 4.87, N, 4.38; HRMS calcd for MH^+: 310.1074, Found: 310.1068.

Compound **20b**

^{1}H NMR (600 MHz, $CDCl_3$) δ 7.43 (1 H, d, *J* = 8.4 Hz), 7.08 (1 H, t, *J* = 7.8 Hz), 7.03 (1 H, d, *J* = 1.8 Hz), 7.98-7.95 (2 H, m), 6.84 (1 H, d, *J* = 8.4 Hz), 6.82 (1 H, d, *J* = 9.0 Hz), 6.24 (1 H, s), 3.72 (3 H, s), 3.53 (3 H, s), 2.38 (3 H, s); ^{13}C NMR (150 MHz, $CDCl_3$) δ 170.6, 160.6, 159.4, 153.9, 151.9, 148.1, 136.6, 129.3, 125.2, 122.6, 121.1, 118.0, 116.6, 115.0, 114.5, 113.9, 55.4, 38.4, 18.8; Anal calcd for $C_{19}H_{17}NO_4 \cdot 1/5H_2O$: C, 69.98, H, 5.79, N, 4.11, Found: C, 69.91, H, 5.95, N, 3.97; HRMS calcd for MH^+: 324.1230, Found: 324.1221.

Compound **21a**

^{1}H NMR (600 MHz, DMSO-*d*$_6$) δ 10.84 (1 H, s), 7.77 (1 H, d, *J* = 9.0 Hz), 7.70 (1 H, td, *J* = 7.2 Hz, *J* = 1.8 Hz), 7.65 (1 H, dd, *J* = 8.4 Hz, *J* = 1.8 Hz), 7.63-7.59 (1 H, m), 7.38 (1 H, d, *J* = 9.6 Hz), 7.35 (1 H, td, *J* = 7.5 Hz, *J* = 1.2 Hz), 2.41 (3 H, s); ^{13}C NMR (150 MHz, DMSO-*d*$_6$) δ 163.4, 160.1, 158.9 (d, *J* = 247.5 Hz), 153.6, 153.2, 142.1, 133.1 (d, *J* = 7.5 Hz), 130.1 (d, *J* = 1.5 Hz), 126.1, 124.8 (d, *J* = 3.0 Hz), 124.5 (d, *J* = 13.5 Hz), 116.4 (d, *J* = 21.0 Hz), 115.7, 115.6, 112.7, 106.2, 18.9; Anal calcd for $C_{17}H_{12}FNO_3$: C, 68.68, H, 4.07, N, 4.71, Found: C, 68.65, H, 4.16, N, 4.74; HRMS calcd for MH^+: 298.0874, Found: 298.0866.

Compound **21b**

^{1}H NMR (600 MHz, $CDCl_3$) δ 7.44 (1 H, d, *J* = 9.0 Hz), 7.40 (1 H, t, *J* = 7.8 Hz), 7.31-7.27 (1 H, br), 7.10 (1 H, t, *J* = 7.8 Hz), 7.05 (2 H, br), 6.86 (1 H, t, *J* = 7.8 Hz), 3.52 (3 H, s), 2.38 (3 H, s); ^{13}C NMR (150 MHz, $CDCl_3$) δ 166.5, 160.5, 158.1 (d, *J* = 249.0 Hz), 153.7, 151.8, 146.6, 132.0(d, *J* = 9.0 Hz), 129.7 (d, *J* = 3.0 Hz), 125.0, 124.6 (d, *J* = 16.5 Hz), 124.6 (d, *J* = 4.5 Hz), 122.5, 118.5, 115.9 (d, *J* = 21.0 Hz), 115.2, 114.8, 37.8, 21.2; Anal calcd for $C_{18}H_{14}FNO_3$ (+1/7 H_2O): C, 68.78, H, 4.60, N, 4.46, Found: C, 68.99, H, 4.70, N, 4.35; HRMS calcd for MH^+: 312.1030, Found: 312.1028.

Compound **22a**

^{1}H NMR (600 MHz, DMSO-*d*$_6$) δ10.94 (1 H, s), 7.84 (1 H, d, *J* = 1.8 Hz), 7.76 (1 H, d, *J* = 8.4 Hz), 7.64 (1 H, dd, *J* = 9.0 Hz, *J* = 1.8 Hz), 7.63 (1 H, dd, *J* = 7.2 Hz, *J* = 1.8 Hz), 7.59 (1 H, dd, *J* = 8.4 Hz, *J* = 1.2 Hz), 7.53 (1 H, td, *J* = 7.8 Hz, *J* = 1.2 Hz), 6.30 (1 H, s), 2.41 (3 H, s); ^{13}C NMR (150 MHz, DMSO-*d*$_6$) δ 165.5, 160.1, 153.6, 153.2, 142.2, 136.4, 131.6, 130.0, 129.8, 129.1, 127.4, 126.1, 115.6, 115.6, 112.7, 106.1, 18.1; Anal calcd for $C_{17}H_{12}ClNO_3$: C, 65.08, H, 3.86, N, 4.46, Found: C, 64.97, H, 4.01, N, 4.53.

Compound **22b**

^{1}H NMR (600 MHz, CD_2Cl_2, 243 K) δ 7.41 (1 H, d, *J* = 8.4 Hz), 7.22-7.17 (3 H, m), 7.12 (1 H, td, *J* = 8.4 Hz, *J* = 1.8 Hz), 7.04 (1 H, d, *J* = 1.8 Hz), 7.02 (1 H, dd, *J* = 8.4 Hz, *J* = 2.4 Hz), 6.18 (1 H, s), 3.48 (3 H, s), 2.31 (3 H, s); ^{13}C NMR (150 MHz, CD_2Cl_2, 248 K) δ 167.5, 160.3, 153.3, 152.3, 145.9,

135.9, 130.4, 130.3, 129.6, 128.9, 126.8, 125.2, 122.5, 118.5, 114.9, 114.8, 37.0, 18.8; Anal calcd for $C_{18}H_{14}ClNO_3$: C, 65.96, H, 4.31, N, 4.27, Found: C, 65.96, H, 4.45, N, 4.28; HRMS calcd for MH^+: 328.0735, Found: 328.0728.

Compound **23a**
^{1}H NMR (600 MHz, DMSO-*d6*) δ 10.52 (1 H, s), 7.89 (1 H, s), 7.40 (1 H, d, *J* = 9.0 Hz), 7.66 (1 H, d, *J* = 9.0 Hz), 7.62 (1 H, dd, *J* = 7.8 Hz, *J* = 1.8 Hz), 7.52 (1 H, td, *J* = 7.8 Hz, *J* = 1.8 Hz,), 7.19 (1 H, d, *J* = 7.8 Hz), 7.06 (1 H, t, *J* = 7.2 Hz), 3.89 (3 H, s), 2.41 (3 H, s); ^{13}C NMR (150 MHz, DMSO-*d6*) δ 165.4, 160.1, 156.5, 153.7, 153.2, 142.4, 132.4, 129.7, 126.0, 124.8, 120.6, 115.7, 115.3, 112.4, 112.1, 106.1, 56.0, 18.0; Anal calcd for $C_{18}H_{15}NO_4$: C, 69.89, H, 4.89, N, 4.53, Found: C, 70.10, H, 4.95, N, 4.58; HRMS calcd for MH^+: 310.1074, Found: 310.1067.

Compound **23b**
^{1}H NMR (600 MHz, DMSO-*d6*, 60°C) δ 7.60 (1 H, d, *J* = 8.4 Hz), 7.30-7.23 (2 H, m), 7.19 (1 H, s), 7.16 (1 H, d, *J* = 9.0 Hz), 6.90-6.86 (2 H, m), 6.28 (1 H, s), 3.64 (3 H, s), 3.35 (3 H, s), 2.36 (3 H, s); ^{13}C NMR (150 MHz, $CDCl_3$) δ 160.7, 155.4, 153.6, 152.0, 131.1, 128.8, 126.0, 124.6, 122.4, 120.8, 118.1, 114.9, 114.5, 111.0, 55.4, 18.8; Anal calcd for $C_{19}H_{17}NO_4$: C, 70.58, H, 5.30, N, 4.33, Found: C, 70.40, H, 5.30, N, 4.37; HRMS calcd for MH^+: 324.1230, Found: 324.1222.

Compound **24a**
^{1}H NMR (600 MHz, DMSO-*d6*) δ 10.78 (1 H, s), 8.00 (1 H, d, *J* = 1.8 Hz), 7.91 (2 H, t, *J* = 1.8 Hz), 7.91 (1 H, d, *J* = 1.8 Hz), 7.79 (1 H, d, *J* = 9.0 Hz), 7.72 (1 H, dd, *J* = 9.0 Hz, *J* = 1.8 Hz), 6.32 (1 H, d), 2.43 (3 H, s); ^{13}C NMR (150 MHz, DMSO-*d6*) δ 165.3, 160.1, 153.5, 153.2, 142.0, 137.6, 134.5, 131.4, 126.7, 126.0, 116.3, 115.8, 112.8, 106.9, 18.1; Anal calcd for $C_{17}H_{11}Cl_2NO_3$: C, 58.64, H, 3.18, N, 4.02, Found: C, 58.40, H, 3.46, N, 4.15; HRMS calcd for MH^+: 348.0189, Found: 348.0185.

Compound **24b**
^{1}H NMR (600 MHz, $CDCl_3$) δ 7.50 (1 H, d, *J* = 8.4 Hz), 7.29 (1 H, t, *J* = 1.8 Hz), 7.20 (2 H, d, *J* = 1.8 Hz), 7.04 (1 H, d, *J* = 2.4 Hz), 6.97 (1 H, dd, *J* = 8.4 Hz, *J* = 1.8 Hz), 6.29 (1 H, s), 3.51 (3 H, s), 2.41 (3 H, s); ^{13}C NMR (150 MHz, $CDCl_3$) δ 167.8, 160.3, 154.0, 151.8, 146.9, 138.3, 135.1, 130.5, 127.1, 125.7, 122.6, 118.6, 115.4, 114.7, 38.6, 18.8; Anal calcd for $C_{18}H_{13}Cl_2NO_3$ (+1/7 H_2O): C, 59.20, H, 3.68, N, 3.84, Found: C, 59.18, H, 3.94, N, 3.84; HRMS calcd for MH^+: 362.0345, Found: 362.0348.

Compound **25a**
^{1}H NMR (600 MHz, $CDCl_3$) δ 7.58 (1 H, d, *J* = 8.4 Hz), 7.35 (1 H, dd, *J* = 4.8 Hz, *J* = 1.2 Hz), 7.21 (1 H, d, *J* = 1.8 Hz), 7.14 (1 H, dd, *J* = 8.4 Hz, *J* = 2.4 Hz), 6.90 (1 H, dd, *J* = 4.2 Hz, *J* = 1.2 Hz), 6.82 (1 H, t, *J* = 3.6 Hz), 6.32 (1 H, s), 3.49 (3 H, s), 2.45 (3 H, s); ^{13}C NMR (150 MHz, DMSO-*d6*) δ 166.4, 160.2, 1536, 153.2, 142.7, 137.8, 134.5, 133.4, 125.9, 125.6, 116.1, 115.3, 112.5, 106.5, 20.9, 18.1; Anal calcd for $C_{19}H_{17}NO_3$ ·1/7H_2O: C, 70.35, H, 5.29, N, 4.28, Found: C, 70.42, H, 5.43, N, 4.44; HRMS calcd for MH^+: 308.1281, Found: 308.1274.

Compound **25b**
^{1}H NMR (600 MHz, $CDCl_3$) δ 7.44 (1 H, d, *J* = 8.4 Hz), 7.03 (1 H, d, *J* = 2.4 Hz), 6.99 (1 H, dd, *J* = 8.4 Hz, *J* = 2.4 Hz), 6.94 (2 H, s), 6.92 (1 H, s), 6.24 (1 H, s), 3.51 (3 H, s), 2.39 (3 H, s), 2.18 (6 H, s); ^{13}C NMR (150 MHz, $CDCl_3$) δ 171.3, 160.7, 153.8, 152.0, 148.2, 137.9, 135.4, 132.1, 126.4, 125.1, 122.6, 117.8, 114.8, 114.5, 38.4, 21.3, 18.8; Anal calcd for $C_{20}H_{19}NO_3$: C, 74.75, H, 5.96, N, 4.36, Found: C, 74.88, H, 6.27, N, 4.39; HRMS calcd for MH^+: 322.1438, Found: 322.1431.

Compound **26b**
^{1}H NMR (600 MHz, $CDCl_3$) δ 7.65 (1 H, d, *J* = 8.4 Hz), 7.17 (1 H, d, *J* = 2.4 Hz), 7.14 (1 H, dd, *J* = 8.4 Hz, *J* = 2.4 Hz), 6.20 (1 H, s), 3.28 (3 H, s), 2.47 (3 H, s), 2.26 (1 H, s), 1.68 (4 H, t, *J* = 13.2 Hz), 1.59 (4 H, m), 1.19 (2 H, m); ^{13}C NMR (150 MHz, $CDCl_3$) δ 176.2, 160.4, 154.2, 151.9, 147.4, 125.9, 123.2, 119.3, 115.6, 115.4, 41.8, 37.6, 29.6, 25.6, 25.6, 18.9; Anal calcd for $C_{18}H_{21}NO_3$ ·1/6H_2O: C, 71.36, H, 7.12, N, 4.62, Found: C, 71.44, H, 7.21, N, 4.66; HRMS calcd for MH^+: 300.1594, Found: 300.1588.

Compound **27b**

^{1}H NMR (600 MHz, $CDCl_3$) δ 8.06 (1 H, d, *J* = 8.4 Hz), 7.81 (1 H, d, *J* = 7.8 Hz), 7.76 (1 H, s), 7.57 (1 H, t, *J* = 8.4 Hz), 7.50 (1 H, d, *J* = 8.4 Hz), 7.28 (4H, br), 7.08 (1 H, br), 6.19 (1 H, s), 3.59 (3 H, s), 2.30 (3 H, s); Anal calcd for $C_{22}H_{17}NO_3 \cdot 1/6H_2O$: C, 76.15, H, 5.06, N, 4.04, Found: C, 76.17, H, 5.26, N, 4.17; HRMS calcd for MH^+: 344.1281, Found: 344.1273.

Compound **28b**

^{1}H NMR (600 MHz, $CDCl_3$) δ 7.94 (1 H, d, *J* = 1.2 Hz), 7.75 (2 H, d, *J* = 8.4 Hz), 7.65 (1 H, d, *J* = 8.4 Hz), 7.50 (1 H, td, *J* = 6.6, 1.2 Hz), 7.46 (1 H, td, *J* = 6.6, 1.2 Hz), 7.36 (1 H, d, *J* = 8.4 Hz), 7.34 (1 H, dd, *J* = 8.4, 1.2 Hz), 7.11 (1 H, d, *J* = 1.8 Hz), 6.95 (1 H, dd, *J* = 8.4, 2.4 Hz), 6.21 (1 H, s), 3.59 (3 H, s), 2.33 (3 H, s); ^{13}C NMR (150 MHz, DMSO-d_6) δ 170.8, 160.5, 154.0, 151.9, 148.1, 133.9, 132.8, 132.6, 129.6, 128.9, 128.0, 127.8, 127.7, 126.7, 125.2, 122.9, 118.0, 115.0, 114.5, 38.6, 25.7, 18.7, 18.0; Anal calcd for $C_{22}H_{17}NO_3 \cdot 1/6H_2O$: C, 76.15, H, 5.06, N, 4.04, Found: C, 76.17, H, 5.26, N, 4.17; HRMS calcd for MH^+: 344.1281, Found: 344.1272.

Compound **29b**

^{1}H NMR (600 MHz, $CDCl_3$) δ 8.31 (1 H, s), 7.73 (1 H, td, *J* = 7.8 Hz, *J* = 1.8 Hz), 7.68 (1 H, d, *J* = 7.8 Hz), 7.46 (1 H, d, *J* = 9.0 Hz), 7.23 (1 H, t, *J* = 6.0 Hz), 7.07 (1 H, d, *J* = 9.0 Hz), 6.98 (1 H, s), 6.24 (1 H, s), 3.57 (3 H, s), 2.39 (3 H, s); Anal calcd for $C_{17}H_{14}N_2O_3$ (+1/6 H_2O): C, 68.54, H, 4.87, N, 9.40, Found: C, 68.55, H, 4.98, N, 9.21.

Compound **30b**

^{1}H NMR (600 MHz, $CDCl_3$) δ 7.58 (1 H, d, *J* = 8.4 Hz), 7.26 (1 H, s), 7.17 (1 H, d, *J* = 2.4 Hz), 7.10 (1 H, dd, *J* = 8.4 Hz, *J* = 2.4 Hz), 6.47 (1 H, dd, *J* = 3.0 Hz, *J* = 1.2 Hz), 6.32 (1 H, d, *J* = 1.2 Hz), 6.30 (1 H, m), 3.48 (3 H, s), 2.45 (3 H, s); ^{13}C NMR (150 MHz, DMSO-d_6) δ 160.5, 159.5, 154.1, 151.9, 147.5, 147.1, 144.7, 125.5, 122.9, 118.9, 117.4, 115.3, 115.1, 111.4, 38.5, 18.9; Anal calcd for $C_{16}H_{13}NO_4$: C, 67.84, H, 4.63, N, 4.94, Found: C, 67.54, H, 4.68, N, 4.96; HRMS calcd for MH^+: 284.0917, Found: 284.0916.

Compound **31b**

^{1}H NMR (600 MHz, DMSO-d_6) δ 10.56 (1 H, s), 7.94 (1 H, d, *J* = 1.2 Hz), 7.76 (2 H, s), 7.58 (2 H, s), 7.26 (1 H, s), 6.29 (1 H, s), 2.42 (3 H, s), 2.37 (6 H, s); ^{13}C NMR (150 MHz, $CDCl_3$) δ 163.1, 160.4, 154.1, 151.8, 147.6, 137.5, 132.4, 130.9, 127.0, 125.7, 123.7, 119.2, 116.0, 115.5, 39.0, 18.9; Anal calcd for $C_{16}H_{13}NO_3S$: C, 64.20, H, 4.38, N, 4.68, Found: C, 64.20, H, 4.66, N, 4.78; HRMS calcd for MH^+: 300.0689, Found: 300.0691.

Crystal Data for Compounds 7a and 7b

	Compound **7a**	Compound **7b**
Recrystan Solvent	$CHCl_3$ / MeOH	AcOEt
Formula	$C_{17}H_{13}NO_3$	$C_{18}H_{15}NO_3$
Crystal system	Orthorhombic	Monoclinic
Space group	*P*bcn	$P2_1/c$
Unit cell dimensions	a = 8.0923(6) Å<br>b = 13.0253(9) Å<br>c = 24.0566(17) Å	a = 25.0823(19) Å<br>b = 8.4430(6) Å<br>c = 14.3193(11) Å<br>β= 106.2670(10)°..
Z	8	8
Final R indices [I>2sigma(I)]	R_1 = 0.0399, wR_2 = 0.0943	R_1 = 0.0448, wR_2 = 0.1001
CCDC No.	2014199	2014197
